# Comparative mitogenomics of freshwater snails of the genus *Bulinus*, obligatory vectors of *Schistosoma haematobium*, causative agent of human urogenital schistosomiasis

**DOI:** 10.1038/s41598-022-09305-7

**Published:** 2022-03-30

**Authors:** Si-Ming Zhang, Lijing Bu, Lijun Lu, Caitlin Babbitt, Coen M. Adema, Eric S. Loker

**Affiliations:** 1 Department of Biology, Center for Evolutionary and Theoretical Immunology, University of New Mexico, Albuquerque, NM 87131 USA; 2 Parasitology Division, Museum of Southwestern Biology, University of New Mexico, Albuquerque, NM 87131 USA

**Keywords:** Evolution, Genetics, Zoology, Diseases

## Abstract

Among the snail genera most responsible for vectoring human-infecting schistosomes, *Bulinus, Biomphalaria, and Oncomelania*, the former is in many respects the most important. Bulinid snails host the most common human blood fluke, *Schistosoma haematobium,* responsible for approximately two-thirds of the estimated 237 million cases of schistosomiasis. They also support transmission of schistosomes to millions of domestic and wild animals. Nonetheless, our basic knowledge of the 37 *Bulinus* species remains incomplete, especially with respect to genome information, even including mitogenome sequences. We determined complete mitogenome sequences for *Bulinus truncatus*, *B. nasutus*, and *B. ugandae*, and three representatives of *B. globosus* from eastern, central, and western Kenya. A difference of the location of *tRNA-Asp* was found between mitogenomes from the three species of the *Bulinus africanus* group and *B. truncatus*. Phylogenetic analysis using partial *cox1* sequences suggests that *B. globosus* is a complex comprised of multiple species. We also highlight the status of *B. ugandae* as a distinct species with unusual interactions with the *S. haematobium* group parasites deserving of additional investigation. We provide sequence data for potential development of genetic markers for specific or intraspecific *Bulinus* studies, help elucidate the relationships among *Bulinus* species, and suggest ways in which mitogenomes may help understand the complex interactions between *Schistosoma* and *Bulinus* snails and their relatives.

## Introduction

Of the world's 237 million estimated cases of human schistosomiasis^1^, about 85% occur in sub-Saharan Africa^2–4^, and approximately two-thirds of the patients are afflicted with urogenital schistosomiasis^2,3^. Like intestinal schistosomiasis, urogenital schistosomiasis causes underappreciated morbidity along with well-known pathological symptoms such as hematuria, and an association with bladder pathology including cancer^5–7^. Damage to the urogenital system, especially in females, is increasingly recognized as a factor favoring the transmission of the Human Immunodeficiency Virus (HIV)^8,9^. The relatively recent emergence of urinary schistosomiasis in Corsica, France^10,11^ has highlighted the opportunistic nature of *Schistosoma haematobium*, particularly with respect to a large number of recent studies suggestive of its ability to hybridize with closely related species like *S. bovis* or *S. curassoni*^12–14^.

Freshwater pulmonate snails of the genus *Bulinus* (Gastropoda, Planorbidae) are the obligate intermediate hosts of *S. haematobium*. Thirty-seven species of *Bulinus* are recognized and predominantly distributed on the African continent including Madagascar and associated smaller oceanic islands, several Mediterranean islands and southern continental Europe, and southwest Asia including the Arabian Peninsula^15^. Several species including *Bulinus globosus*, *B. nasutus*, *B. truncatus,* and *B. africanus* serve as intermediate hosts of *S. haematobium*. Additionally, bulinids also vector other parasites. *Bulinus forskalii* serves as an intermediate host for *S. guineensis*, and *B. globosus* transmits *S. intercalatum*, both schistosome species being etiologic agent of human intestinal schistosomiasis^16^. Several *Bulinus* species are implicated in transmission of other members of the *S. haematobium* group of species which cause schistosomiasis in domestic and wild animals^17–22^. Moreover, bulinids can also serve as intermediate hosts for other trematode species, particularly amphistomes, pathogens of livestock^23,24^. Clearly, *Bulinus* snails play a crucial role in transmission of snail-transmitted diseases in the tropical world.

Better understanding all aspects of the biology of vector snails including their population genetics, distributions, basic immunobiology including susceptibility to infection, symbionts and co-infections, and response to environmental change are critical to develop sound strategies for the future control of human schistosomiasis and other snail-transmitted diseases of concern. One of the basic, long-standing challenges is how to accurately and efficiently identify *Bulinus* species. Characters based solely on morphological traits are often difficult to discern and subject to eco-phenotypic variation. Molecularly-based approaches have revealed that considerable genetic variation exists within and among species and have been essential in providing a framework of objective criteria for more rigorously delineating taxa^25–27^. Molecular data including partial DNA sequences from mitochondrial cytochrome c oxidase subunit 1 (*cox1*) and internal transcribed spacer regions (ITS) of nuclear ribosomal DNA (rDNA) have been provided to address questions related to population genetics and phylogenetics^26,28–30^. These studies have provided much-needed insights into phylogenetic relationship among species but have not always yielded consistent results^26–28,31^. More comprehensive molecular data for *Bulinus* snails are needed.

Complete mitochondrial genome (mitogenome) sequences have proven to be useful in addressing a broad range of questions in evolutionary biology^32,33^. Mitogenomes have been reported for several molluscan species, including multiple species of *Biomphalaria*^34–36^ and one species of *Oncomelania*^37^. For the genus *Bulinus*, only a mitogenome sequence for *B. truncatus* has thus far been reported^38^. This is surprising because of the magnitude of the problems posed by *S. haematobium* and related species. To help fill this gap, we applied high through-put Illumina sequencing to determine six complete mitogenomes for *Bulinus* snails, five specimens collected in Kenya and one from laboratory-reared *Bulinus truncatus,* originally from Egypt. The study provides basic sequence information for designing more markers for identifying bulinid species and revealing relationships among species, highlights some specific topics deserving additional study pertaining to understanding compatibility and biogeography of snails and schistosomes, and provides new tools of use in studies of host use, transmission, and control of human schistosomiasis.

## Results

**Figure 1:**
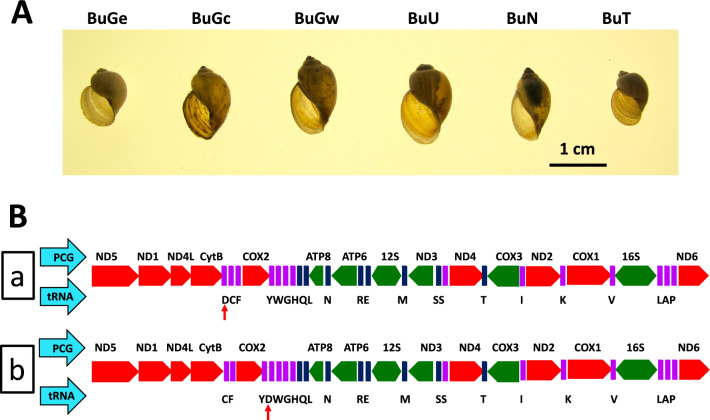
Shell morphology of six snail specimens (**A**) and the arrangement of protein-coding genes (PCG) and tRNAs in six mitogenomes (**B**). In **B**, diagram **a** shows the gene arrangement of the mitogenome of *B. truncatus*, whereas **b** displays the gene arrangements of the mitogenomes of *B. globosus*, *B. nasutus*, and *B. ugandae*. Note the different position of tRNA-Asp (red arrow). The abbreviations BuGe, BuGc, and BuGw refer to three specimens of *B. globosus* that were collected from eastern, central, and western Kenya, respectively. BuT, BuN, and BuU represent *B. truncatus*, *B. nasutus*, and *B. ugandae*, respectively. The same abbreviations are applied to all figures below and in supplementary Table 1.

All six bulinid mitogenomes were comprised of 37 genes, including 13 protein-coding genes (PCG), 22 transfer RNA (tRNA) genes and 2 ribosomal RNA (rRNA) genes. Location of the genes in the six mitogenomes was conserved, with the only difference noted being the location of tRNA-Asp of *B. truncatus* relative to the other three species (*B*. *nasutus*, *B. ugandae*, and *B. globosus*) (Fig. 1). The GenBank accession numbers of the six mitogenomes generated from *B. truncatus, B*. *nasutus*, *B. ugandae*, and three specimens of *B. globosus* from eastern, central, and western Kenya, are MK414449, MK414450, MK414451, MK414452, MK414453 and MK414454, respectively. More detailed information on gene organization for all six mitogenomes is provided in the Supplementary Table S2 and Supplementary Fig. S1.

**Figure 2:**
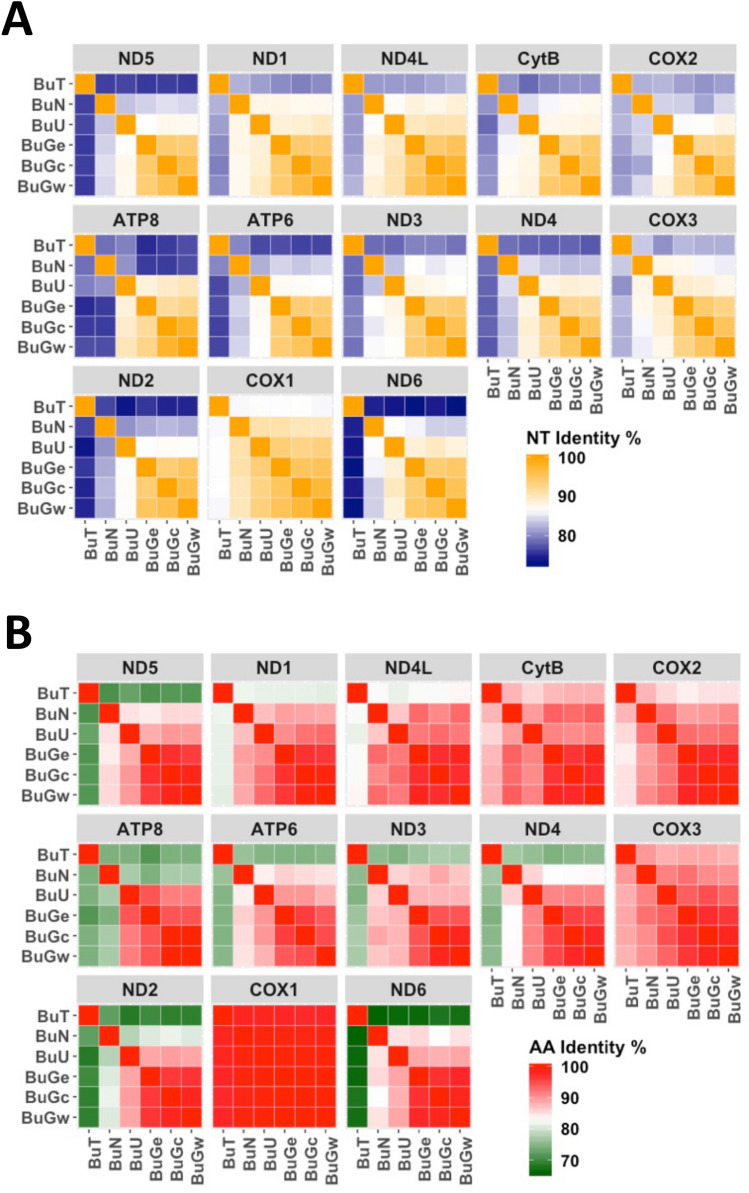
Heatmaps showing the degree of identity of nucleotides (nt) (**A**) and amino acids (aa) (**B**) between species/specimens.

Heatmap analyses revealed that the most conserved gene and its gene-product (protein) was *cox1* whereas *atp8* showed a relatively high degree of variation (Fig. 2).

**Figure 3:**
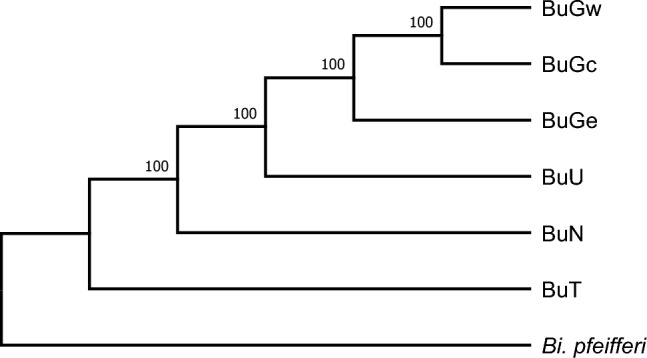
A maximum likelihood (ML) phylogenetic tree with 1000 bootstrap replicates of full-length mitochondrial genome nucleotide sequences of *Bulinus* species. All bootstrap values are indicated at supported nodes.

Built on the full length mitogenome sequences, a phylogenetic analysis revealed that *B. globosus* from eastern and western Kenya are divergent. In our samples, *B. ugandae* was the species most closely related to *B. globosus* (Fig. 3). As expected, *B. truncatus*, a member of *B. truncatus/tropicus* complex^15^, was most distantly related to the other three *Bulinus* species (all members of the *B. africanus* species group)^15^. This result was also supported by organization of gene order of the mitogenomes (Fig. 1).

**Figure 4:**
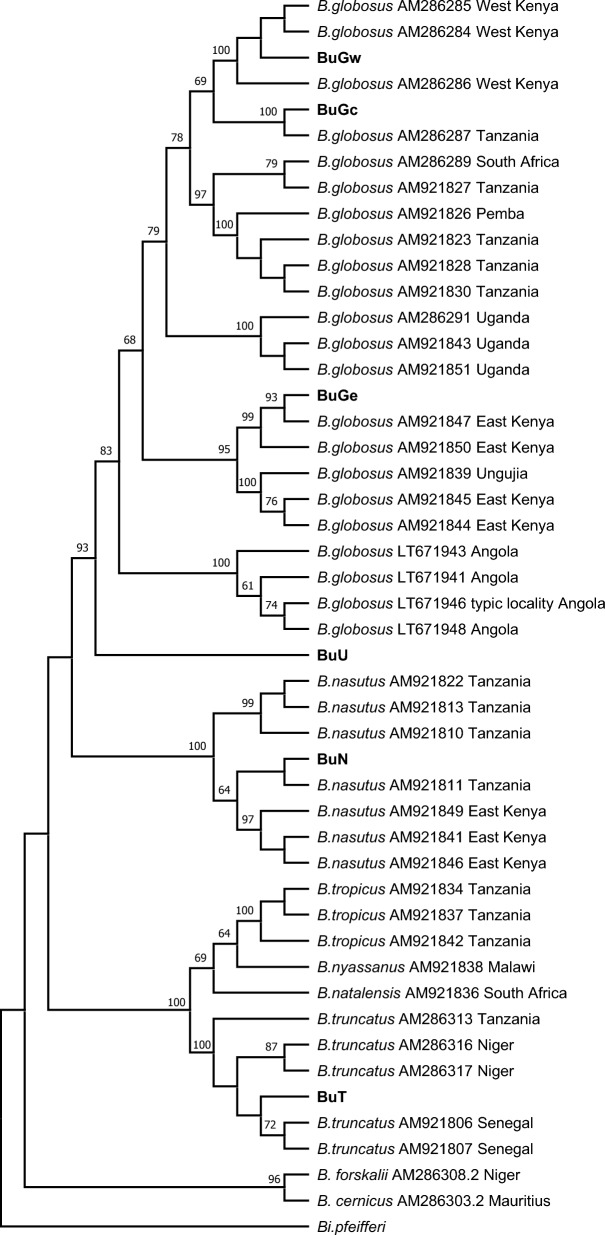
A ML phylogenetic tree with 1000 bootstrap replicates generated using partial *cox1* gene sequences. Bootstrap values > 60 are indicated at supported nodes. Publicly available sequences utilized in this analysis are indicated by GenBank accession number.

Since partial *cox1* sequences have been documented for many *Bulinus* species, we compared *cox1* sequences from our samples with those previously reported. The phylogenetic analysis shows that the four species used in this study, representing two of the four generally elaborated *Bulinus* species groups, fit well within the framework of known species already reported (Fig. 4). This analysis further highlights the considerable diversity inherent in what might be referred to as a *B. globosus* species complex and the presence of a distinct, but sometimes overlooked species, *B. ugandae,* often associated with Lake Victoria.

## Discussion

Thirty-seven species of freshwater pulmonate snails of the genus *Bulinus* have been divided into four species groups: *Bulinus forskalii* group (11 species), *Bulinus truncatus/tropicus* group (14 species), *Bulinus africanus* group (10 species), and *Bulinus reticulatus* group (2 species)^15^. Definitive evolutionary relationships among the four species groups and delimitations of species within each group, especially within the *africanus* species group, have remained elusive.

For example, there is considerable confusion regarding differentiation of *B. globosus* and *B. africanus*^15^. Differences in the male copulatory organs between the two species have been noted, with the penis sheath of *B. africanus* being longer and broader than the preputium, as compared to *B. globosus*^15,39^. Later studies, however, suggested that such characters are not reliable for species discrimination^40^. Previous studies using limited molecular data have retrieved contradictory results. Morgan et al. (2002) grouped *B*. *africanus* with *B. nasutus* rather than with *B. globosus* based on analysis of partial ITS sequences^41^. Another study using combined *cox1* and ITS sequences indicated *B. africanus* is clustered within a clade of *B. globosus*^26^.

**Figure 5:**
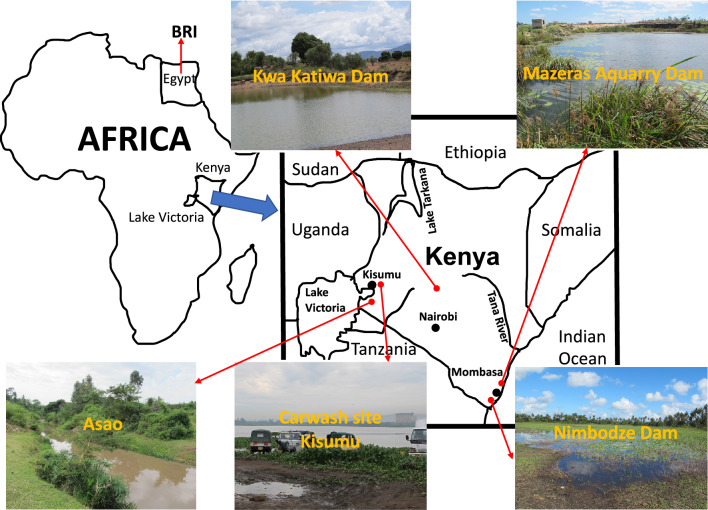
Localities of snail samples used for the study. The maps were drawn by the first author S-MZ and photos were taken by S-MZ.

In the present study, we collected *B. globosus* samples from eastern, central, and western Kenya (Fig. 5). At the beginning of the work, we suspected the sample from Asao stream, a perennially flowing stream in western Kenya might be *B. africanus*. As stated by Brown et al. (1981), *B. africanus* is widely distributed in Kenya and associated with perennially flowing streams and permanently filled dams^42^. However, our phylogenetic trees based on *cox1* sequences clearly showed the west Kenya sample from Asao stream closely grouped with *B*. *globosus* samples collected from Kisumu, about 15 km away^26^ (Fig. 4). In addition, *cox1* sequence of a snail sampled from the eastern coastal area of Kenya clustered with sequences reported as *B. globosus* by others from eastern Kenya (Fig. 4). The sample from central Kenya was more closely related to those from west Kenya and Tanzania. We observed samples from eastern and western Kenya to be divergent, in agreement with findings based on microsatellite data^31,43^. Notably, our study also supports that *B*. *globosus* from Angola, the type locality of *B. globosus*^15^, is divergent from *B. globosus* from other localities^31^ (Fig. 4). According to these molecular data, it is likely that there are multiple disparate lineages represented as “*B. globosus*”, one from the type locality, and conservatively, at least two additional related lineages represented in Kenya alone. Pennance (personal communication), who has collected additional relevant mitogenome sequences, has also confirmed the notion of a *B. globosus* “species complex” (see also^31,43^). Whether these lineages should be classified into different formally named species or subspecies, or simply highlight the existence of a single broadly distributed and highly variable species is an interesting question deserving resolution. It bears directly on the issue of defining the full spectrum of snail host species for *S. haematobium* as *B. globosus* in its various guises is repeatedly implicated as an excellent host for *S. haematobium* and several other related schistosomes^15,20^. More molecular evidence such as mitogenomes collected from a wide range of geographic locations will help resolve the question.

*Bulinus ugandae* is found in Lake Victoria and associated backwaters, and north to South Sudan and Ethiopia. It appears to be refractory to infection with *S. haematobium*^42^, but is host for *S. bovis*, a parasite infecting livestock^44^. Our sample collected from the Kisumu shoreline of Lake Victoria is more closely related to *B. globosus* than to the other species we sampled. This agrees with a previous ITS sequence-based study suggesting that *B. ugandae* is closely related to *B. globosus*^41^.

*Bulinus nasutus*, a species repeatedly implicated in *S. haematobium* transmission in coastal East Africa, including Kenya^45^, was collected from a reservoir in southern Mombasa, a coastal city of eastern Kenya. This specimen and those reported from eastern Kenya and Tanzania by others are well clustered. As a member of the *B. africanus* group, *B*. *nasutus* also grouped with *B. globosus* and *B. ugandae* (*africanus* group) (Fig. 4)**.**

*Bulinus nasutus*, another member of the *B. africanus* group, also as supported by Figs. 3 and 4, has been repeatedly implicated in *S. haematobium* transmission in coastal East Africa, including Kenya^45^. Our *cox1* analysis is suggestive of variation within this taxon as well, that may also have implications with respect to *S. haematobium* transmission.

*Bulinus truncatus* originating from Egypt has been maintained with NIH support for decades, most recently at the Biomedical Research Institute (BRI), Maryland, USA, and is used for routine maintenance of the life cycle of *S. haematobium* (www.afbr-bri.com). Unlike the preceding three taxa we examined which were all from Kenya and belong to the *Bulinus africanus* group, the *B. truncatus* specimen is classified to the *Bulinus truncatus/tropicus* group, supported by both the analysis of the whole mitogenome sequences (Fig. 3) and the *cox1* phylogenetic analysis (Fig. 4). In addition, the position of *tRNA-Asp* revealed in this study is also different from that of *B. africanus* group (Fig. 1B).

The mitogenome sequence characterized by us is equal in length (13,767 bp) to the sequence characterized independently from *B. truncatus* also obtained from BRI^38^. The two mitogenomes generated by two different sequencing methods (Nanopore^38^ vs Illumina) differ by 7 nucleotides (0.05%); resulting in 5 non-synonymous replacements (one each in *nad5* and *nad3*, three in *atp6*), one synonymous replacement in the start codon of *nad4* and a single pyrimidine (T, C) transition in the 16S rDNA sequence. These differences indicate a modest level of genetic diversity in the BRI stock of *B. truncatus.*

It is noteworthy that the *B. truncatus nad4* gene does not have a regular TAA stop codon observed from *nad4* in other *Bulinus* species, nor does it have an incomplete stop codon punctuated by a downstream tRNA sequence. Secondary structures, however, may mark gene boundaries in polycistronic mitochondrial RNA^46^. Annotation indicated that the last complete trinucleotide codon of the 3’ terminus of the *nad4* gene sequence is followed by a single T nucleotide and, immediately downstream, by an inverted repeat. This TAACAGAATTCTGTTA sequence likely yields a hairpin structure in the 3’ end of the gene transcript to define an incomplete stop codon (T—), that is completed by polyadenylation of the mRNA.

A reliable taxonomy of the genus *Bulinus* and useful genetic markers for species identification are a fundamental prerequisite for fully understanding the epidemiology of *Bulinus*-transmitted schistosomiasis in humans and animals. Our study provides basic data to design primers for further in-depth studies: markers derived from different mitogenome regions can be developed for studies with objectives ranging from species differentiation (*cox1*) to sequences like *atp8* which may be appropriate for studies of intraspecific variation. As next generation sequencing (NGS) becomes even more cost-effective further comparative analysis of complete mitogenomes will follow^47,48^ including, as noted by Pennance (personal communication) to allow novel insights into relationships and evolution among *Bulinus* species.

The perspective offered by more mitogenomes will be particularly important for the gastropod family Planorbidae because, in addition to containing two genera of medically relevant snails (i.e., *Bulinus* and *Biomphalaria*), a third genus, *Indoplanorbis*, hosts the representatives of the Asian *Schistosoma indicum* group of veterinary significance^49,50^. *Indoplanorbis* is generally considered the sister genus to *Bulinus*^27,41^ and given the support from the fossil record for an African origin for *Bulinus*^50^, it has been considered that *Bulinus*-like snails originating in Africa may have dispersed to Asia and given rise to *Indoplanorbis* which increasing evidence suggests is actually a complex of as many as five cryptic species^49–52^. This is turn supports the notion that *Bulinus*-transmitted *Schistosoma* may have recolonized Asia using *Indoplanorbis* as their snail host^50,52^. A greater representation of mitogenomes from *Bulinus*, along with needed mitogenome sequences from the various cryptic species of *Indoplanorbis* would provide further insight into the *Bulinus*-*Indoplanorbis* connection and its relation to today’s distribution of species of *Schistosoma*.

Additionally, the relationships between the *Schistosoma mansoni* species group (transmitted by *Biomphalaria*) and the *S. haematobium* species group (transmitted by *Bulinus*) in sub-Saharan Africa remain enigmatic, particularly given that the two snail genera involved are not close relatives within the Planorbidae, and the arrival of *Biomphalaria* to Africa is believed to have been relatively recent, long after *Bulinus* had originated there^41^. More precise knowledge of host use, systematic clarification of the snail groups involved, and provision of more genomic information, to which this study contributes, will help to us appreciate the manner in which African *Schistosoma* diversified.

It has been noted that gastropods display an unusually large variety of gene orders among their mitogenomes^46,53,54^. Within the order Hygrophila, complete mitogenomes have been published for representatives of only three of eight families, the Planorbidae^34–36,38,55^, Lymnaeidae^56–58^, and Physidae^59^. For *Bulinus*, recognized by Bouchet et al. (2017) as a member of a separate family^60^, the Bulinidae, mitochondrial gene order is almost identical to representatives of the Planorbidae for which mitogenomes are available. Several phylogenetic studies also group *Bulinus* within the Planorbidae, and a role in transmission of *Schistosoma* parasites is also suggestive of inclusion of *Bulinus* in the Planorbidae^27,41,61^. Planorbidae mitochondrial gene order is similar to the Lymnaeidae except for differences in location of a few tRNAs, whereas gene order is quite different in the Physidae^60^. Further studies are needed to characterize more gastropod mitogenomes, which will shed light on their evolution and diversification in mollusks and at the same time, provide more genetic markers for population genetics, evolutionary and phylogenetic studies.

## Materials and methods

### Specimens

*Bulinus nasutus* was collected from Nimbodze dam (03° 28.32ʺ S, 39° 27.08ʺ E), southern Mombasa, Kenya. *B. ugandae* was sampled from the car-wash site in Kisumu city, Kenya (00° 05′ 45.00ʺ S, 34° 44′ 57.69ʺ E). Three *B. globosus* snails were collected from Mazeras quarry dam (03° 54ʹ 0.58ʺ S, 39° 31ʹ 0.72ʺ E) (northern Mombasa, eastern Kenya), Kwa Katiwa Dam (01° 12ʹ 0.00ʺ S, 37° 16ʹ 0.41ʺ E) (central Kenya), and Asao (00° 19′ 5.50ʺ S, 35° 0′ 24.99ʺ E) in western Kenya, in 2013 (Figs. 1 and 5). *Bulinus truncatus* was obtained from Biomedical Research Institute (BRI); it had originally been collected from Egypt decades ago (http://www.afbrbri.com/schistosomiasis/materials-offered/) (Figs. 1 and 5). The field collected specimens were placed in 90% ethanol and stored at 4 °C until use. This study was undertaken with the approvals of the National Commission for Science, Technology, and Innovation (permit number P/15/9609/4270 and P/21/9648), National Environmental Management Authority (permit numbers NEMA/AGR/46/2014 and NEMA/AGR/149/2021), and Kenya Wildlife Service (permit numbers KWS 0004754 and KWS-0045-03-21).

### Extraction of DNA

After removing the shell, the whole body of a single live snail was ground to a fine powder using mortar and pestle in liquid nitrogen. The powder was transferred to 1.5 ml tubes for subsequent DNA extraction using a Qiagen Miniprep kit-based mtDNA enrichment method^36,62^. For ethanol-preserved samples, the ethanol was replaced by distilled water. The water was changed every 12 h for 4 times. After removing shells, individual snails were placed in a 1.5 ml tube and ground using a pestle and a CTAB method was used for DNA extraction^63^.

After extraction, DNA samples were treated with RNAse A (Invitrogen) at 37 °C for 30 min and then 70 °C for 10 min. DNA samples were further purified using SPRselect Beads (Beckman Coulter). Quality and quantity of DNA were measured using a Nanodrop spectrophotometer and Qubit fluorometer (Invitrogen).

### Preparation, amplification, and sequencing of Illumina libraries

A genomic library for each sample was prepared (KAPA Hyper Prep Kit, KAPA Biosystems, www.kapabiosystems.com). Each snail DNA was barcoded with an adaptor. The libraries were sequenced (150 nucleotide (nt) × 2 paired-end) on the Illumina NextSeq500 platform at the UNM Biology Department’s Molecular Biology Facility (http://ceti.unm.edu/core-facilities/molecular-biology.html)^36^.

### Assembly and annotation of mitogenomes

Two complementary methods were used to assemble mitochondrial genomes, reads baiting and iterative mapping assembly using MITOBIM^64^ and de novo assembly using SPAdes^65^. MITOBIM is a tool developed to map reads to a related reference mitogenome and then use these to recursively find additional reads to build the mitogenome of the target. The complete *Biomphalaria glabrata* mitochondrion genome (NCBI accession NC_005439.1) was used to bait *Bulinus* mitochondrial reads for genome assembly and extension until a saturation status was reached, where no further reads were found to extend the genome assembly. The longest contig generated with SPAdes with BLASTN e-value < 10^–5^ against the reference mitogenome of the closely related snail *Biomphalaria glabrata* (*Bulinus* and *Biomphalaria* belong to Planorbidae)^36^was selected as the mitogenome assembly. Output from the two methods were aligned and manually checked for consistency to generate the mitogenome sequence for each sample. To check the read support consistency, reads were mapped to the final assembled mitogenomes and visualized using Integrated Genome Viewer^66^.

The annotation of mitogenomes was conducted using MITOS2 that also includes an updated protein identification model^47,67^. Both RefSeq 63 Metazoa and RefSeq 81 Metazoa, were used as reference for verification and confirmation of gene predictions. Otherwise, MITOS2 default settings were applied (E-value exponent: 2; Final maximum overlap: 50; fragment quality factor: 100). Moreover, we re-checked mitogenome sequences manually using the ExPASY translate tool (http://web.expasy.org/translate/) and did BLAST to confirm the reading frames of protein coding genes. Final annotation was based on criteria from Fourdrilis et al. (2018) that incorporate unique aspects of mitogenome biology, including transcription as polycistronic RNA, the punctuation model and completion of stop codons by polyadenylation of mRNA transcripts^68^.

### Genetic and phylogenetic analysis

Sequence alignments and percent identity of nucleotides (nt) and amino acids (aa) were determined using Clustal Omega^69^. Heat-maps of pair-wise sequence identities were generated using R package ggplot2^70^. All the intermediate data organization and filtering were done with in-house bash and Perl scripts and Microsoft Excel.

Phylogenetic analyses for full mitochondrial genomes and partial *cox1* were done using the maximum likelihood method and conducted with MEGAX^71,72^. *Biomphalaria pfeifferi* sequences were utilized as the outgroup for both analyses. For both datasets the GTR + G + I model was selected by Akaikeʼs information criterion in MEGAX^72^. Both analyses were conducted with 1000 bootstrap replicates.

## Supplementary Information

Supplementary Figure 1.

Supplementary Table 1.

## Data Availability

GenBank accession numbers of the mitogenomes for *B. truncatus, B*. *nasutus*, *B. ugandae*, and three specimen of *B. globosus* from eastern, central, and western Kenya, are MK414449, MK414450, MK414451, MK414452, MK414453, and MK414454, respectively.
