# Author Correction: Comparative mitogenomics of freshwater snails of the genus *Bulinus*, obligatory vectors of *Schistosoma haematobium*, causative agent of human urogenital schistosomiasis

**DOI:** 10.1038/s41598-025-97357-w

**Published:** 2025-04-17

**Authors:** Si-Ming Zhang, Lijing Bu, Lijun Lu, Caitlin Babbitt, Coen M. Adema, Eric S. Loker

**Affiliations:** 1 Department of Biology, Center for Evolutionary and Theoretical Immunology, University of New Mexico, Albuquerque, NM 87131 USA; 2 Parasitology Division, Museum of Southwestern Biology, University of New Mexico, Albuquerque, NM 87131 USA

Correction to: *Scientific Reports* 10.1038/s41598-022-09305-7, published online 30 March 2022

The original version of this Article contained an error in the legend of Figure 1, where the descriptions for **a** and **b** in panel **B** were switched.

As a result, the legend of Figure 1:

“Shell morphology of six snail specimens (**A**) and arrangement of protein-coding genes (PCG) and tRNAs of six mitogenomes (**B**). In part **B**, diagram **a** shows the gene arrangements of the mitogenome of *B. globosus*, *B. nasutus* and *B. ugandae* whereas **b** shows the mitogenome of *B. truncatus*. Note the different position of tRNA-Asp (red arrow). The abbreviations of BuGe, BuGc and BuGw are three specimens of *B. globosus* that were collected from eastern, central, and western Kenya, respectively. BuT, BuN, and BuU represent *B. truncatus*, *B. nasutus*, and *B. ugandae*, respectively. The same abbreviations are applied to all figures below and supplementary Table 1.”

now reads:

“Shell morphology of six snail specimens (**A**) and the arrangement of protein-coding genes (PCG) and tRNAs in six mitogenomes (**B**). In **B**, diagram **a** shows the gene arrangement of the mitogenome of *B. truncatus*, whereas **b** displays the gene arrangements of the mitogenomes of *B. globosus*, *B. nasutus*, and *B. ugandae*. Note the different position of tRNA-Asp (red arrow). The abbreviations BuGe, BuGc, and BuGw refer to three specimens of *B. globosus* that were collected from eastern, central, and western Kenya, respectively. BuT, BuN, and BuU represent *B. truncatus*, *B. nasutus*, and *B. ugandae*, respectively. The same abbreviations are applied to all figures below and in supplementary Table 1.”

The original Article has been corrected.

