# Supplementary figures and images for: Comparative mitogenomics of freshwater snails of the genus Bulinus, obligatory vectors of Schistosoma haematobium, causative agent of human urogenital schistosomiasis
Source: Sci Rep. 2022 Mar 30;12:5357. doi: 10.1038/s41598-022-09305-7 (PMC8967911; doi:10.1038/s41598-022-09305-7)

Supplementary Figure 1.

Maps of six mitogenomes of *Bulinus* snails

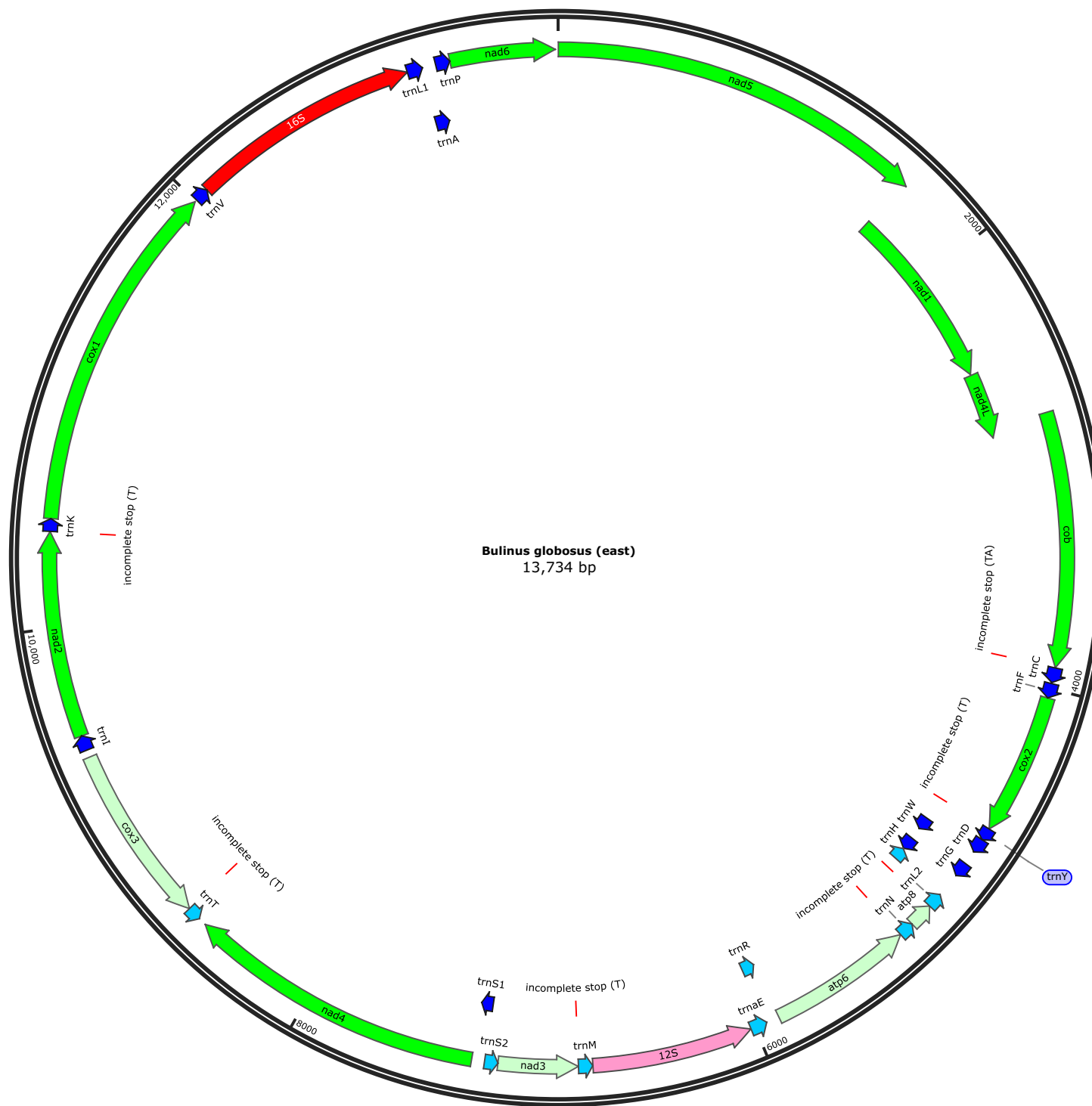

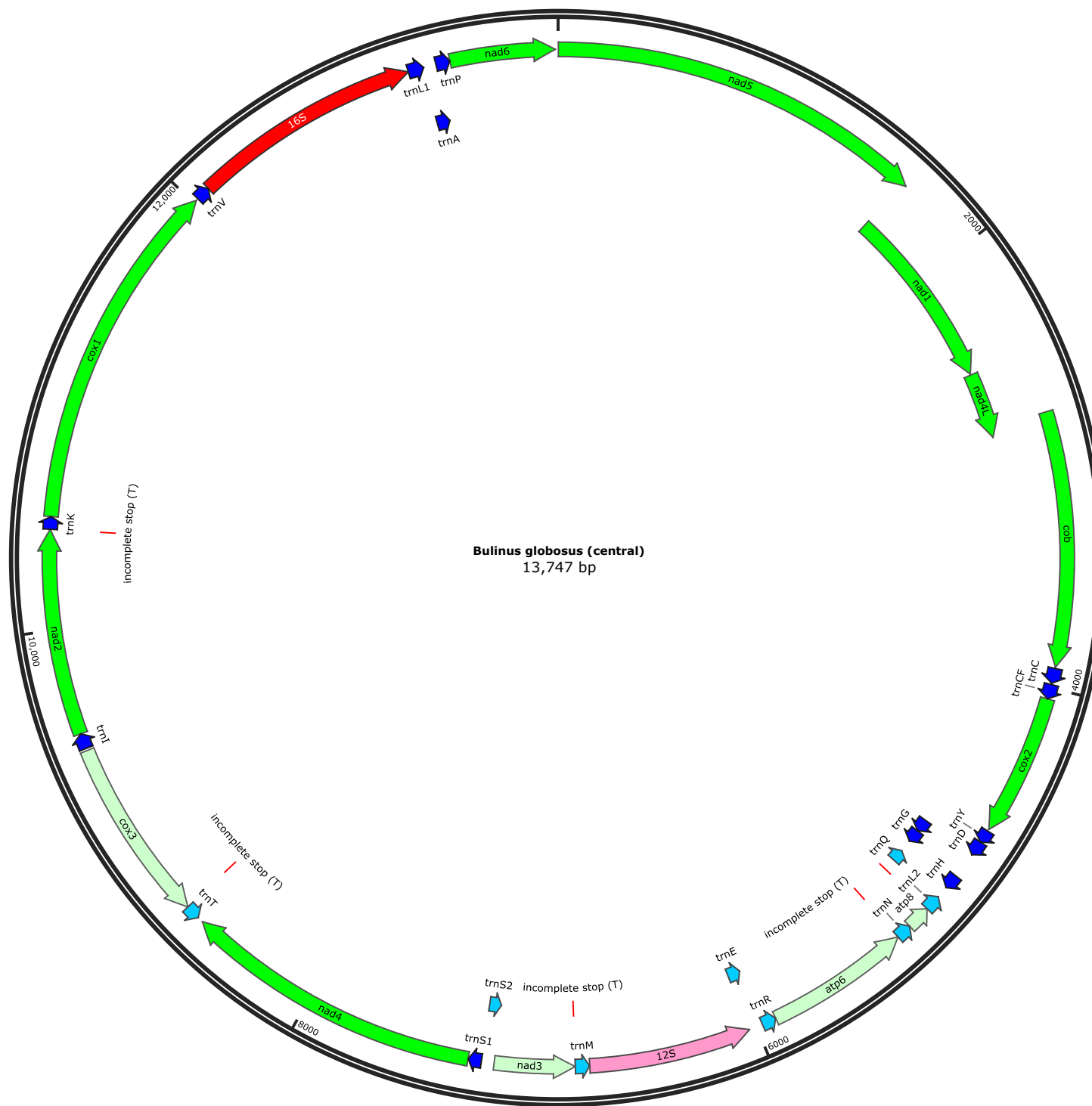

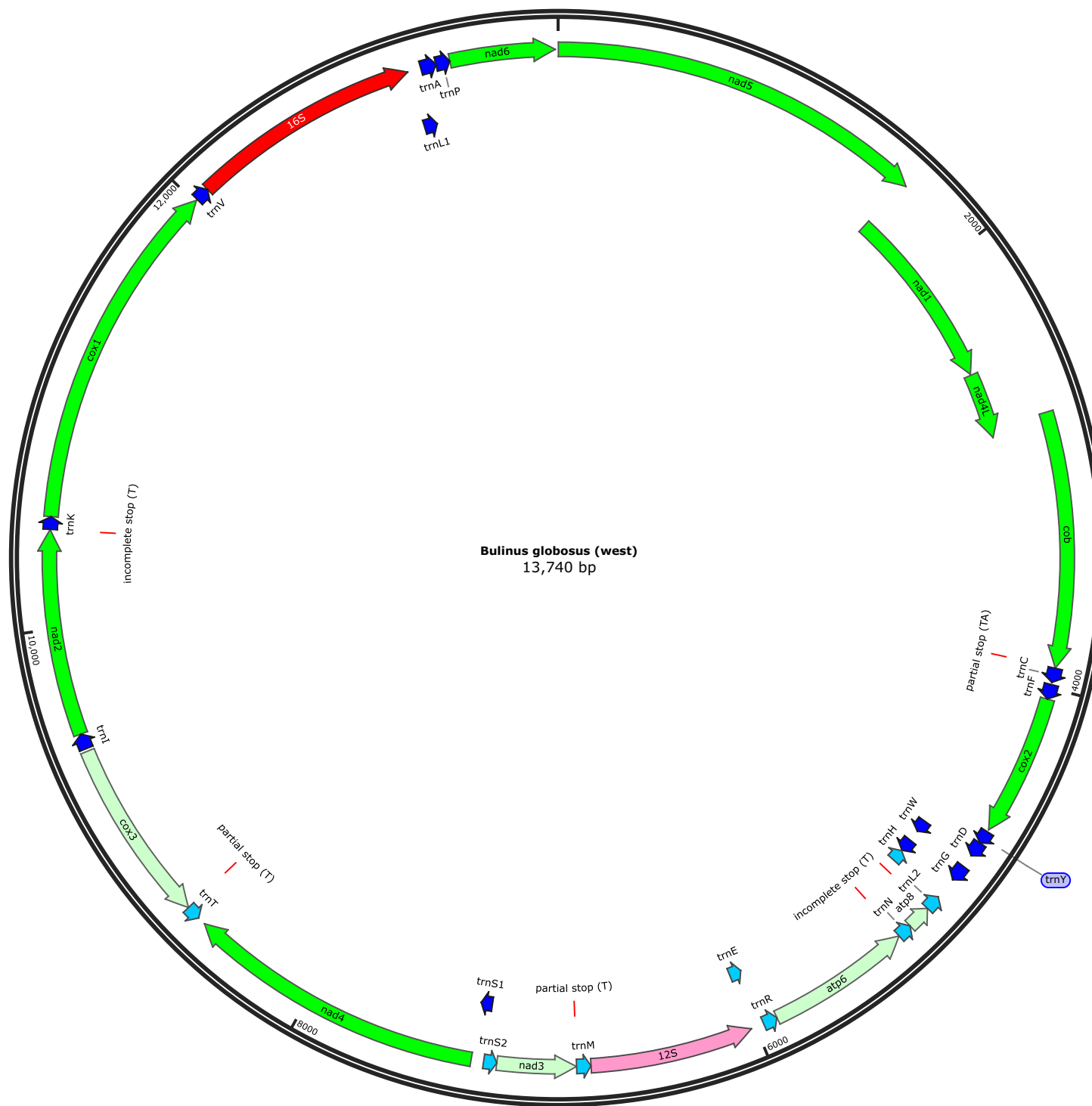

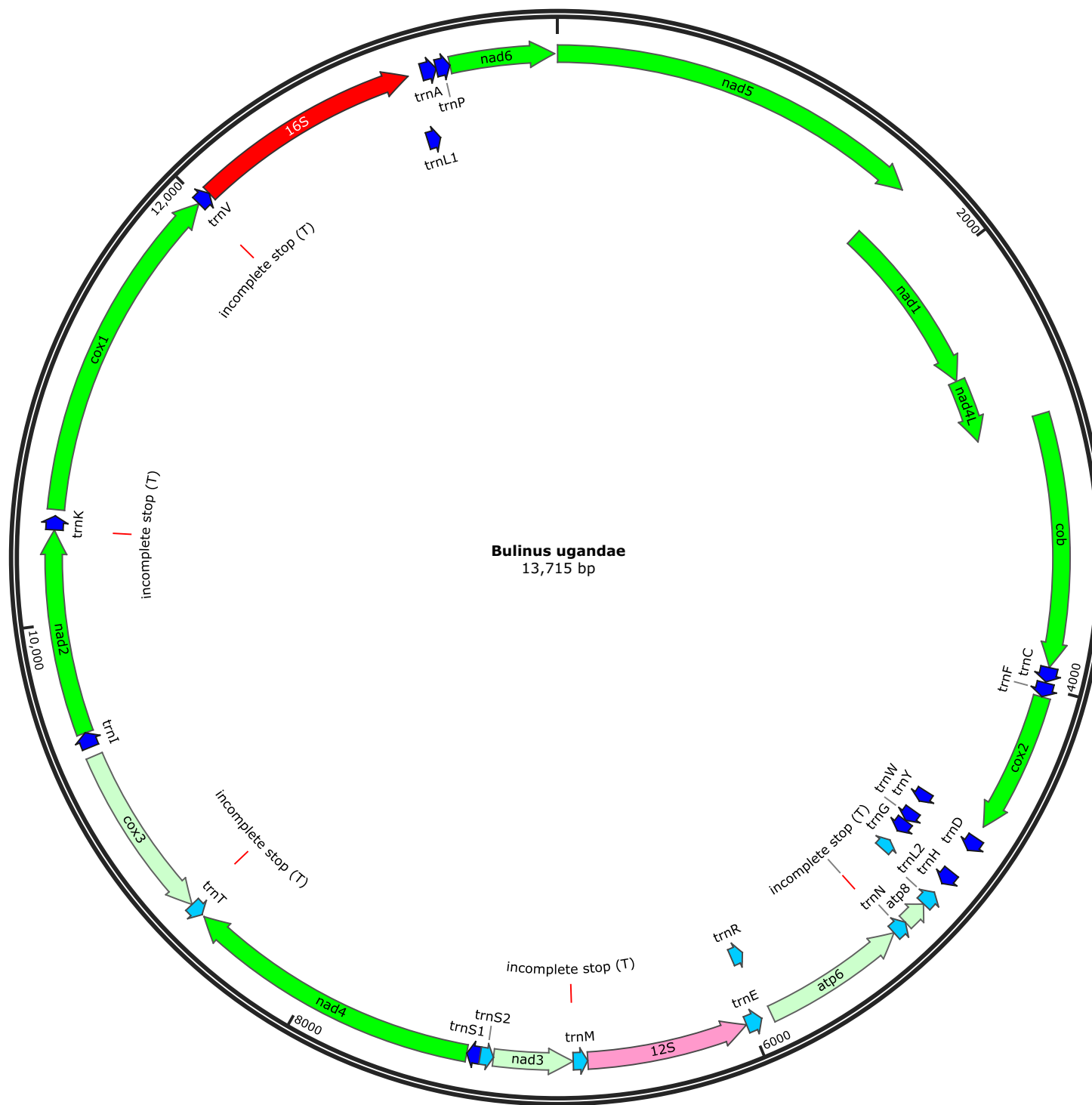

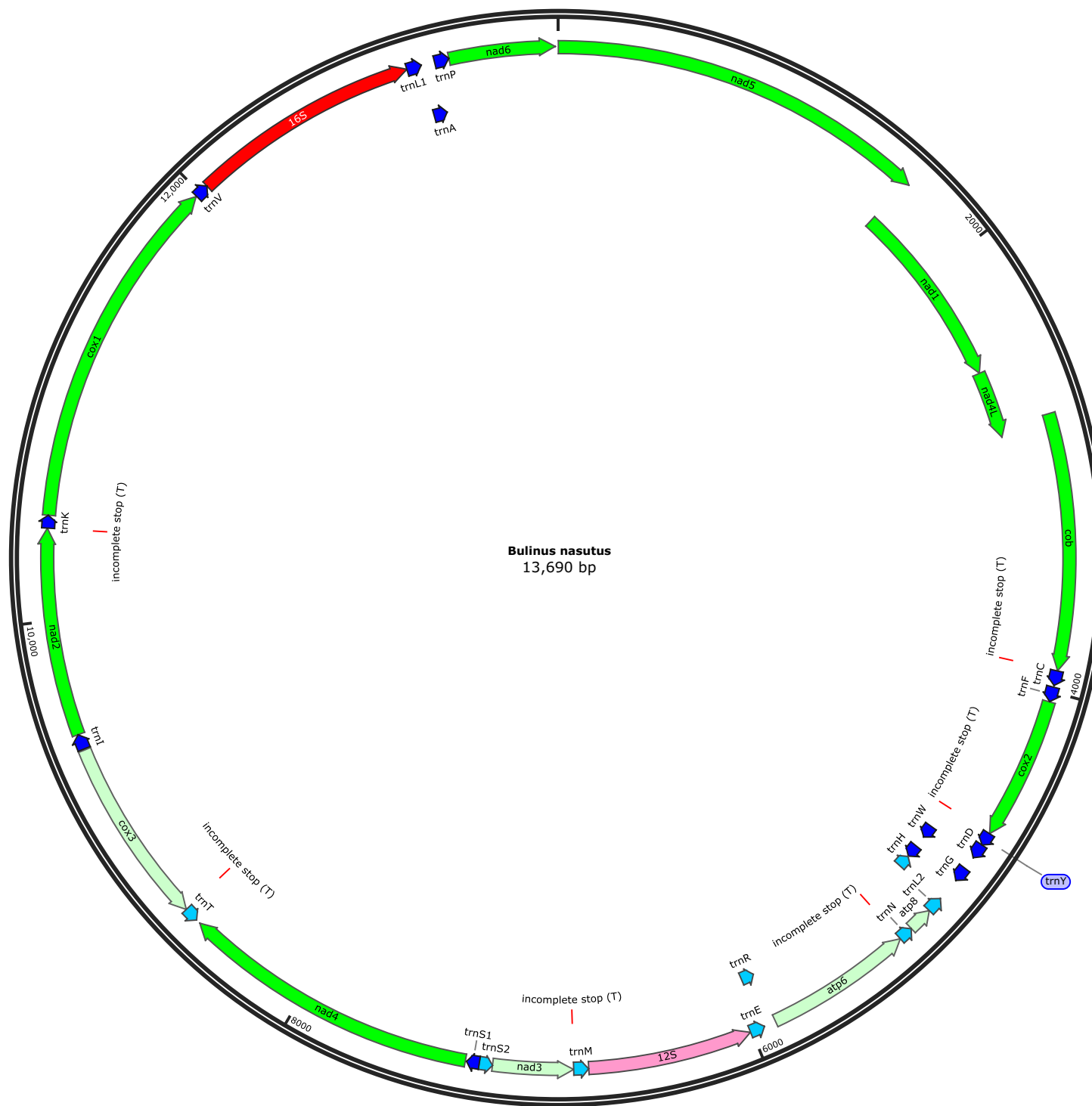

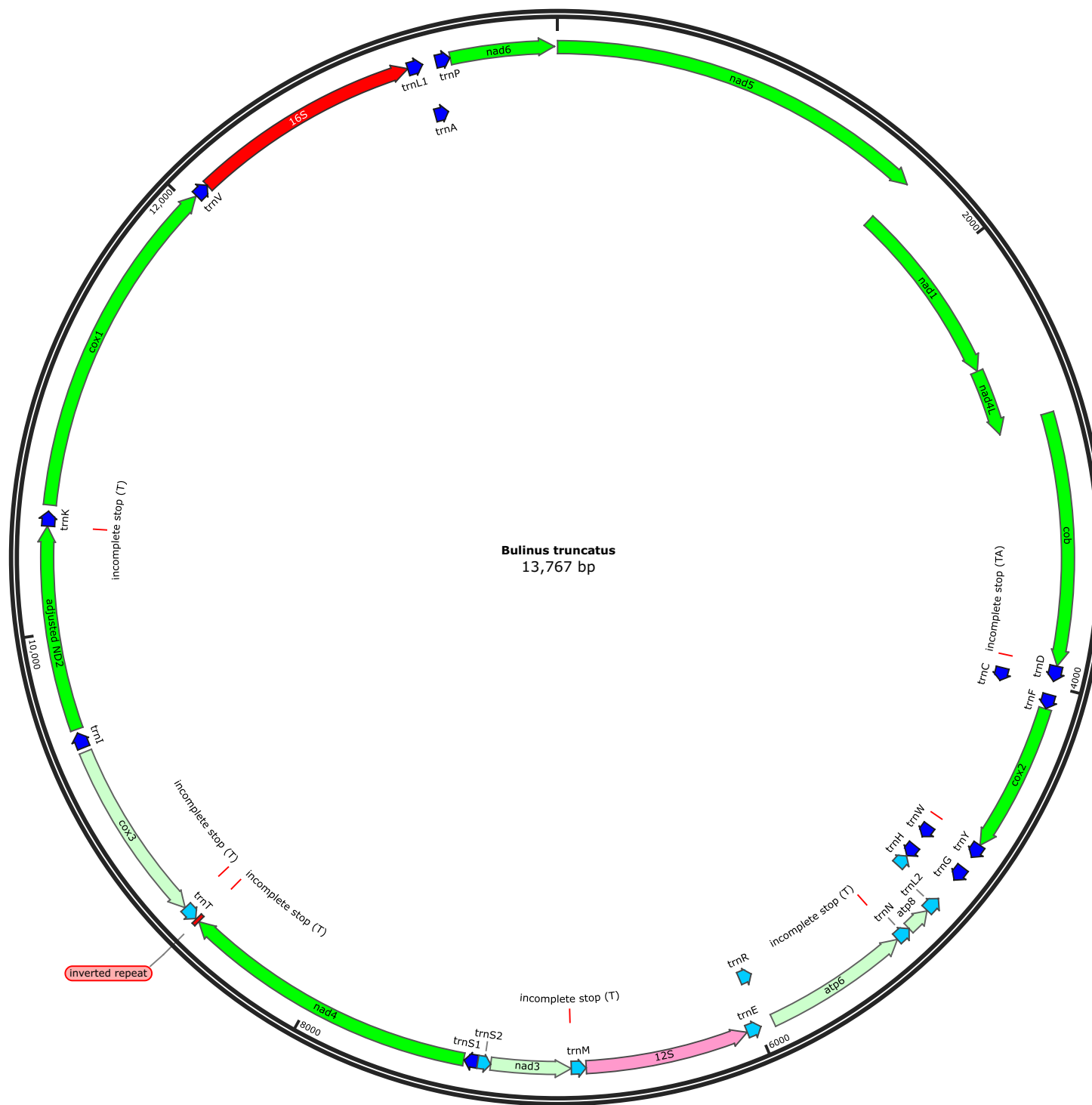

Supplement: Supplementary file 1 — Supplementary Figure 1. [file 41598_2022_9305_MOESM1_ESM.pdf]
